# Address before the Alumni Association

**Published:** 1871-06

**Authors:** James Truman


### Selections.

---

### ADDRESS BEFORE THE ALUMNI ASSOCIATION.

BY JAMES TRUMAN, D. D. S.

"What contributes most to professional power?" It is a question that each and all must analyze, and, if possible, endeavor to reach a solution. We all profess, or should profess to believe that knowledge is power, or, in other terms, that he wields the greatest force in the world whose education has been the most perfect. While this is true, and almost axiomatic in its truthfulness, the modes to be adopted, the means to subserve the great end in view, are by no means so clearly defined. In no branch of human training have we cleared a path entirely free from difficulties, and we can not claim an exception in favor of our own profession. Young in years, still encumbered with crude ideas, inseparable from this period of experience, we have largely developed in our professional brain and overweening self-consciousness that we have accomplished very much for our boyish age. But the boy is growing into the man. As the garments are being changed for adult life, there comes the knowledge that the limit of our information is circumscribed to a degree that borders on the ridiculous. Time is fast verging into the half century since the profession of dentistry may be said to have been born. It had a life long anterior to this, but it was crude, misshapen, simple in its aims and narrow in its cultivation. Slowly the child has matured, yet rapidly, when the difficulties that have surrounded its growth are taken into consideration. The stepping stones in its history are plainly marked and can be read by all. We see the progress, but we can not realize the difficulties that have environed each step; the hours of anxiety, of hard labor, of deep study, that have formed out of chaos the beautiful structure we now behold. * * *

When I recall the early days of dental education and compare them with those of the present, I feel that progress has truly marked our career. When I remember the names of a Harris, a Wildman, an Arthur, a Maynard, in the great work of regeneration, the question, "What contributes most to professional power?" is answered, and it might be given in one word, *education*. These men, as well as a host of others, have builded that others might reap, have given of their intellectual force that you and I are enabled to perform works worthy of our generation. But, if we profit not by their example, we will have no future worth the record.

I do not belong to that class of mind that stands ever ready to ignore the past and the benefits derived from it; but it is the ever living present we now have to deal with, and the needs of that near future that will hold us accountable if we fail to do the work of the hour. Are we, then, performing our whole duty? This each must answer for himself, but to my mind there are many things yet to be done, and it is the purpose of this brief address to point out some of these.

When medical and dental colleges were organized in this country, the importance of a thorough preliminary education was felt and considered; but owing to the peculiar condition of the country, this could never be made an absolute requirement before entering a school. The country was new, it required professional men in large numbers to meet the wants of the different communities, and it was therefore left to the individual to judge of his own fitness to commence the career he had chosen. The result has been in many respects injurious. We see it painfully manifest in the medical profession, where the dead weight of ignorance cripples the energies of the more enlightened, and makes American medicine a by-word and reproach in Europe. In dentistry the same results in degree have been reached. In the earlier years the strong, natural ingenuity and practical ability of our countrymen, enabled them to take a leading position. They accomplished miracles, and were and are the wonder of the world in this department; and because they have done this many are deceived into the belief that any further advance in education is of very little if any value. Here lies our great peril. Self-complacency has destroyed individuals, has sapped the life of nations, and ended in their total destruction. The result can not be different with our profession. It is only by an humble appreciation of our great needs, of our fearful short-comings, that we can hope still further to progress and keep in the vanguard of the advancing hosts.

We have passed through the early period of our professional life. The cultivation of the mechanical details absorbed, and properly so, the minds of the best thinkers and workers in our midst. This was the great need. Without this, theory was valueless; indeed, without it the profession itself ran in danger of becoming a monster of malpractice. But we have given our years of best thought in this direction. We had almost perfected the mechanical branch when vulcanite, with its quacks, came as an avalanche upon us, threatening to overwhelm all that was good in this direction, and to destroy the labors of half a century. How much injury this has accomplished is known to you all, and I need not dilate upon it. The operative branch has had its trials, its days of amalgams and plastic fillings, but the good sense and true faith of the many have so far stood as a wall of adamant against the insiduous approaches of these fell destroyers. Over all these, operative dentistry has advanced with rapid strides, until it would seem that there can be but little left to wish for. We have learned to build our foundations well, and are no longer in doubt in regard to the result of the majority of our operations. Having, therefore, carried the two main branches of our profession to a point at which they can be left to that gradual growth and improvement that years will bring, are we not prepared for greater efforts in other departments?

The distinct propositions I would then submit to your consideration are these:

1st. Is a preliminary examination necessary before entering the study of dentistry?

2d. Should the time of study be lengthened?

3d. Is it necessary to enlarge the knowledge of collateral branches?

4th. Are compulsory laws, requiring a grade of ability, expedient?

I am fully aware of the difficulties that meet me at the outset in the consideration of the first query. Some of these have been already adverted to; but in the consideration of a question, the difficulties to be encountered are not to be taken into account. Is the path to be pursued a proper one? This decided upon, there is but one course to pursue, and that an unswerving devotion to its attainment. It must be apparent to every reflecting observer, that no structure of any value can be reared upon an insecure foundation. As this is true in architecture, so it is equally true in mental culture. The man who begins to study at the top of the ladder finds himself, instead of advancing, to be ever, crab-like, crawling backward in the laborious effort to find the first round. That the labor is a discouraging one, needs no assertion to those who have tried it. Now, is not this the exact position of a large proportion of medical and dental students to-day, and especially of dental? This is not said with a view to discourage any laudable ambition. Far be it from me to do this. I would rather encourage every earnest effort to advance. While the profession was in its pupilage, this was not wholly detrimental; but now it is working a very great injury, indeed, is the drag that must land us in a wilderness of crudities, if a strong arm is not interposed to check it. Our colleges have done much, indeed, they have accomplished wonders, as is evidenced to your own comprehension, but they can not do all. Now what are the facts? Men untrained in special studies, often with meagre education, enter our colleges and are met at the outstart with problems the most difficult, requiring a large amount of intellectual culture to comprehend, much more to master. They are overwhelmed at once. They are at the top and vainly essay to discover the beginning. The result is a constant floundering through insurmountable difficulties until the end. Is it any wonder that men are turned out of colleges, good in practice it may be, and even passably good in theory, yet wholly unfitted to cope with that intellectual power, that is striding through all lands, and demands the fullest and completest attainment? There can be no remedy for this evil but a preliminary examination, thorough in its character and arbitrary in its demands. That this must come, whether this year or next, or a score of years hence, is certain, and only through its influence will we be relieved of the stigma of graduating uneducated men.

You can do much to help this sentiment in the profession. I am happy in the belief that the largest proportion of our graduates have been men of sterling attainments; but it is only through the accidents of life that this is so. Many more have not been of this character, a misfortune to them as well as to their alma mater. I speak plainly, for the hour requires plain thoughts.

The subject of education is receiving more attention than ever before. The fearful war, now devastating and making desolate the homes of two nations, has had one good result amidst the fearful physical and moral destruction it has brought about, and that is, that it has opened the eyes of the whole world to the power of an educated people. Already the national legislature is ringing with appeals to make our education a subject of governmental care, and our State is looking toward compelling every parent to send the child to school. Is it not then, proper and meet that we, in convention assembled, should earnestly take into consideration what will best advance our grade of scholarship, and give an impetus to our professional work? That it must be sooner or later done is plain to my mind, that it will be done is equally plain.

Should the time of study be lengthened?

I presume the necessity of this nee ls no argument. It is evident that our colleges are all lapsing into weakness by catering to the current American idea of rapidity of acquirement. The whole system is one of cramming from beginning to end, and while much is learned it requires the constant after training of years to digest that which has been secured. Superficiality is the bane of American life in all departments, and yet even in this land the boy apprentice to a mechanical branch can not enter the position of a journeyman under five years pupilage, and this, perhaps simply to give manipulative dextery, brain work being mainly left for superiors. The dentist, however, is expected to acquire his profession in two or at most three years, and to become to some extent cultivated in many collateral sciences. His first duty is to perfect himself in mechanical dentistry. This is a combination of several branches, and alone should have a close apprenticeship of four or five years. This study will embrace an amount of knowledge from the collateral sciences that will fully occupy the time allotted; but then we have operative dentistry also, that combines within its needs the skill of the artist, the knowledge of the anatomist, the coolness of the surgeon, and the keen insight into the causes and remote results of disease of the general practitioner. That this will require more time than is usually given must be conceded. Then if to these we add the necessary branches of study, anatomy, physiology, chemistry, materia medica, histology and surgery, the curriculum is beyond the management of any single individual in the time given. But these are not all. No dentist can be considered educated who lacks general information on all the sciences. He should have a clear idea of comparative as well as general and special anatomy. If he is deficient in knowledge of the teeth of inferior animals, their general character and minute structure, how can he comprehend those he daily deals with? He can not possibly. Yet how few there are who have any definite comprehension of the intimate relations existing between the structure of the teeth of all animal life. While it is the duty of the college to enter into this study, it can never be fully elaborated in the short time permitted the teacher. Thus I might analyze all the collateral sciences. Each in its turn may be made available for our purpose; if not directly, at least so far as to build up the whole man, and make a cultivated being fit to mingle with and take part with scientific men everywhere. When this is accomplished, then will dentistry receive the full meed of respect, and not until then.

That all this could be achieved in any ordinary pupilage I do not pretend to affirm. I am no advocate for very long periods of probation. I would prefer that an individual should receive the basis of an education and not the whole education; indeed, the latter can never be done. My own opinion is, that four years of college life would be the proper period, following a year of preparatory study in the laboratory. This would give five years of training, a time not too long to be burdensome to a young man willing to be thorough in every department. With such a course, the studies could be properly divided and the labors of the student condensed into digestible proportions. Are these utopian ideas? Not at all. They are fully carried out in Europe, where seven years are required for the study of medicine, following a most severe classical course, and this time is not spent in idleness. To each year is given a study; to one anatomy, another physiology, to another chemistry, and the end of the year must find the student competent to pass a severe examination in each to enable him to advance. At the end of his term he has another severe examination, covering the whole ground; and then a still severer one by the government, in which, if he fails, he is forever prevented from the practice of medicine? Is it any wonder that German science leads the world? If, therefore, we would ever aspire to an equal position, we must reform in this respect, and the sooner we comprehend the importance of this, in all its length and breadth, the more quickly will we be prepared to take measures for its accomplishment.

While in the adolescent period of our profession, immaturity of thought and a lack of attention to more theoretical branches could be overlooked, trusting to the future to cure the evils consequent upon it. Have we reached or are we nearing that future? It seems to me the answer must be clear to every mind that we have. Upon whom, then, devolves the work of regeneration? On you, most assuredly. To you belongs the duty which became yours as part of that diploma, which you so earnestly worked for and so clearly earned. To mould the sentiment of a nation, to make a new path in the world of ideas, is the part of the profound, progressive thinker and worker, and marks the statesman above the mediocracy of his generation. Therefore, to us belongs the same duty, in a minor sense, towards the profession of which we are members. The work is one of magnitude, but difficulties to the morally courageous are but incentives to labor. The isolation of a graduate who settles in a remote section, surrounded by ignorant pretenders, may be imagined, but can hardly be realized. Shall we protect these? Shall the skill acquired by accurate training be wasted in vain endeavors to battle, single handed, this legion of ignorance? I trust not. The remedy, in part, lies at your doors. Organize everywhere in associations. Allow no man to enter who can not show a diploma or submit to a thorough examination. Avail yourselves of your law makers, and secure the future if you can not remedy the past. I am well aware that men of ordinarily good judgment are opposed to any legal force to remove this evil, and while, as a rule, I have more faith in the ultimate spread of intelligence, to arrest this widespread quackery, I have still a belief that the innate depravity of man, or whatever you choose to call it, tempts him to continue the evil, unless superior forces lead into the path of good. At all events, we have the ripe experience of European nations to prove that such laws are of great value, if properly carried out. When a man knows there is but one road to reach the goal desired, he will set about to find that road; but if there be many, he will in all probability select that which seems the shortest, regardless of the many quagmires and obstructions he may meet with. This is human nature. Recognizing this, then, as a fact, our duty is plain. Laws of a stringent character must be passed, prospective and not retrospective. We can not go back and say that every man in practice must submit to an examination; but we can say and must say, that no man in the future shall enter the practice of dentistry without a diploma from a regularly chartered college. This will not benefit *us* materially; but those of a half century hence will feel the full value of our exertions. You graduates of dental colleges can do this. On us this duty devolves, and woe be unto us if we are faithless to our mission.

There is still another duty devolving upon you. Support your dental colleges. See to it that they have material aid that will enable them to carry their mission to its highest fulfillment. Give them the full weight of your moral support and sympathy. The difficulties, the anxieties, the severe labor that falls to the lot of the teachers in these, few know and still fewer appreciate.

There are many critics of instruction, but how few competent to instruct, or if competent are willing to sacrifice self for the good of others. That I believe colleges are imperfect you already know, and that they have performed a wonderful work for good can not be denied. Give, then, the various Faculties your earnest support, and in giving you will build still higher the temple to knowledge.

The hour in which we work is full of promise. Enlarged ideas of man's aims and destiny are taking the place of narrow, bigotted views. The grand in human nature is overleaping the contracted selfishness of by gone ages. Facts are recognized and mere theories discarded. Science, with her irrefutable logic, is scattering the twaddle of the past like chaff before the whirlwind. Men are becoming thinkers, and the more reason guides the helm, the more will the world advance. I congratulate you that in this hour of promise there is so much to be labored for. You know the path, follow it; and though you may not reach all that is desirable of attainment, the fact that your minds have wrought in the intellectual mine, and that you have labored to give the thoughts expression, will bring with it its own true reward. None of us may hope to be appreciated. The true worker never gives this a thought. The development of an idea is to him always self-sustaining. He can wait patiently for results, knowing that in the good time its truthfulness will be manifest. In the love, then, we bear our profession, by the hallowed memories of those who have past—by the interest of the living present, and the great needs of an unborn future, let us ever keep working, and the tiny seed will grow into the sapling and the sapling to the tree, and under its shadowy foliage we may end our years with the consciousness that we have not wholly lived and worked in vain.

